# Use of ketamine for depression and suicidality in cancer and terminal patients: Review of current data

**DOI:** 10.3934/publichealth.2023043

**Published:** 2023-07-21

**Authors:** Aderonke Oyetunji, Christian Huelga, Kailee Bunte, Rachel Tao, Val Bellman

**Affiliations:** 1 University of Missouri Kansas City, Psychiatry Residency Training Program, Kansas City, MO; 2 Kansas City University, College of Osteopathic Medicine, Kansas City, MO; 3 University of Missouri Kansas City, School of Medicine, Kansas City, MO

**Keywords:** ketamine, depression, suicide, cancer, terminal illness

## Abstract

Depression and suicidality are significant challenges faced by cancer patients, particularly those in advanced stages of the disease or nearing the end of life. Conventional antidepressant therapies often have limited effectiveness or delayed onset of action, making the exploration of alternative treatments crucial. The use of ketamine as a potential treatment for depression and suicidality in cancer and terminal patients has gained considerable attention in recent years. This review article aims to provide a comprehensive analysis of the current data regarding the efficacy and safety of ketamine in this specific population. This review presents an overview of clinical trials and case studies investigating the use of ketamine in this population. It explores the effectiveness of ketamine as a standalone treatment or in combination with other interventions. Furthermore, the article addresses the limitations and future directions of research in this field. It highlights the need for larger, well-controlled studies with long-term follow-up to establish the efficacy, safety and optimal treatment parameters of ketamine for depression and suicidality in palliative care.

## 1. Introduction

Patients who have been diagnosed with cancer are three times more likely to develop major depressive disorder (MDD) compared to the general population [1]–[4]. A physician may struggle to diagnose depression in a cancer or terminally ill patient because of a large overlap of symptoms between the two conditions. Insomnia, fatigue, weight loss and changes in appetite can be attributed to a patient's cancer or chemotherapy [5],[6]. However, utilizing an inclusive approach to the criteria of MDD is more sensitive which may lead to increased survival and quality of life for patients with terminal illness and questionable prognosis [6],[7]. Untreated depression can be highly dangerous in a patient with a poor prognosis. When a patient first receives a diagnosis of cancer, they are almost 13 times more likely to commit suicide within seven days than the general population [6]. Traditional antidepressant medications may have limited efficacy in this population. As such, there is a pressing need for alternative treatments that can rapidly alleviate depressive symptoms and reduce the risk of suicide in cancer or palliative care patients. In recent years, a growing body of literature has explored the potential of ketamine as a treatment for depression and suicidality in cancer patients. Despite some promising findings, concerns have been raised about the safety and practical considerations of using ketamine in cancer patients.

Currently, ketamine is reserved as an off-label use for treatment-resistant depression. Physicians may find the rapid onset of ketamine's effects helpful in actively suicidal patients, as current first-line pharmacotherapy options can take several weeks to produce full effect [8],[9]. However, data is somewhat limited regarding the safety and long-term efficacy of ketamine's effects in palliative care and cancer patients. More large-scale clinical trials are needed to determine ketamine's role in depression treatment [9]. Furthermore, ketamine should be studied in combination with chemotherapeutic agents to determine possible drug interactions when utilized for patients with cancer.

The aim of this manuscript is to review the existing literature on the use of ketamine as a treatment for suicidality in cancer patients with a focus on its safety, efficacy and practical considerations. Specifically, the objectives of this manuscript include:

- To provide an overview of the current understanding of the neurobiological mechanisms of ketamine's antidepressant and anti-suicidal effects.
- To evaluate the safety and tolerability of ketamine in cancer patients including the risk of dissociative symptoms, transient increases in blood pressure and heart rate and more serious adverse events such as psychosis and hallucinations.
- To assess the efficacy of ketamine in reducing suicidal ideation in cancer patients including a review of studies that have explored the use of ketamine in this population and their results.
- To discuss practical considerations for the use of ketamine in cancer patients including patient selection, dosing and administration and monitoring of adverse effects.
- To identify gaps in the current literature and highlight areas for future research including the need for larger randomized controlled trials to better understand the safety and efficacy of ketamine in this population.

## 2. Materials and Methods

### 2.1. Literature Search Strategy

A comprehensive search was conducted to identify relevant manuscripts for this literature review. Electronic databases including PubMed, MEDLINE, Embase and PsycINFO were searched using a combination of keywords and medical subject headings (MeSH) related to “ketamine”, “depression”, “suicidality”, “cancer” and “terminal patients”. The search was limited to articles published in English up to February 2023. Additionally, references of relevant articles were hand-searched to identify any additional studies that may have been missed.

### 2.2. Inclusion and Exclusion Criteria

Studies were included in this review if they met the following criteria: (1) focused on the use of ketamine as a treatment for depression and suicidality in cancer and terminal patients, (2) included original research (clinical trials, case series, case reports), (3) reported on outcomes related to depressive symptoms, suicidality, or adverse events, (4) involved human participants and (5) were published in peer-reviewed journals. Studies that solely focused on non-cancer populations or animal models were excluded.

### 2.3. Data Extraction and Synthesis

Two independent reviewers performed the initial screening of titles and abstracts to identify potentially relevant articles. Full-text articles of the selected studies were then retrieved and reviewed for eligibility. Discrepancies were resolved through discussion and consensus. Data were extracted from the included studies using a standardized form which included study characteristics (e.g., author, year, study design), participant characteristics (e.g., sample size, age, cancer type), intervention details (e.g., ketamine dose, administration route), outcome measures and key findings.

### 2.4. Data Analysis

Due to the heterogeneity of the included studies, a narrative synthesis approach was employed to summarize the findings. The results were organized according to the key outcomes such as the efficacy of ketamine in reducing depressive symptoms and suicidality, safety profile and adverse events. The findings were presented descriptively, supplemented with tables or figures when appropriate.

### 2.5. Ethical Considerations

As this review article is based on the analysis of previously published studies, ethical approval was not required.

### 2.6. Limitations

It is important to acknowledge that this review may be subject to publication bias as it focused only on studies published in English. Additionally, the variability in study designs, patient populations and outcome measures across the included studies may limit the generalizability of the findings.

## 3. Results

### 3.1. Definition and properties of ketamine

Ketamine is a dissociative agent that has had a multitude of uses throughout the years. It is able to produce effects within hours by acting as a non-competitive channel blocker of N-methyl-D-aspartate (NMDA) receptors [10]. Ketamine is a dissociative and analgesic that has had sedative uses spanning from veterinary medicine to pediatrics. Others who may have used it as a recreational drug know it as “Special K”, “Kitty” or “vitamin K”. Now, it is beginning to make an appearance in psychiatry.

Phencyclidine, commonly known as PCP, was rejected for use as an anesthetic as a result of its unmanageable and distressing behavioral state [11]. Ketamine was derived from PCP with only 1/10th of the potency, producing a decrease in severe dysphoria and hallucinations which made it a more suitable choice [11]. As far back as the Vietnam War, the U.S. military noted these desirable features along with its quick recovery time, almost immediate analgesic effects and lack of interference with respiratory depression or blood pressure and implemented ketamine as a field anesthetic [12].

### 3.2. Medical uses of ketamine

Ketamine was first approved by the FDA in 1970 as a general anesthetic and has since been used as common practice [13]. In 2019, the S-enantiomer of ketamine, esketamine, was approved as a therapeutic option for treatment-resistant depression [13]. Esketamine can be used intranasally and the isolated S-enantiomer has been shown to have greater affinity for the NMDA-receptor site. When S-ketamine and R-ketamine are compared to one another, S-ketamine produces a more significant analgesic and anesthetic effect and is less likely to induce a psychotic state [14]. Due to these differences, there is emerging research to explore if esketamine may be more suitable for psychiatric uses rather than the racemic mixture. In the U.S, besides being used in anesthesia and as an analgesic, ketamine is used to treat major depressive disorder (MDD) and treatment-resistant depression (TRD) [15]. However, there are off-label uses involving IV ketamine for depression as well as a number of ongoing studies and trials to understand the potential use in post-traumatic stress disorder, anxiety, bipolar disorder as well as drug addictions [15]. Table 1 summarizes the possible ways for ketamine use in palliative care medicine.

**Table 1.** Use of ketamine in palliative care.

**Indications**	**Description**
Perioperative use, dressing changes, orthopedic emergencies.	Single use of IV ketamine often in combination with morphine or midazolam
Pain Management	IV push, IV infusion, subcutaneous infusion for various forms of pain: Neuropathic pain poorly responsive to titrated opioids and oral adjuvant analgesics (eg. antidepressant and/or anticonvulsant) particularly when there is abnormal pain sensitivity - allodynia, hyperalgesia or hyperpathia. Complex ischemic limb pain or phantom limb pain. Poorly controlled incident bone pain (often has a neuropathic element). Complex visceral / abdominal neuropathic pain.
Other	Topical ketamine as an oral rinse has been described to treat mucositis and as a gel for neuropathy.

### 3.3. Mechanism of action for ketamine's antidepressant effects

**Figure 1.**
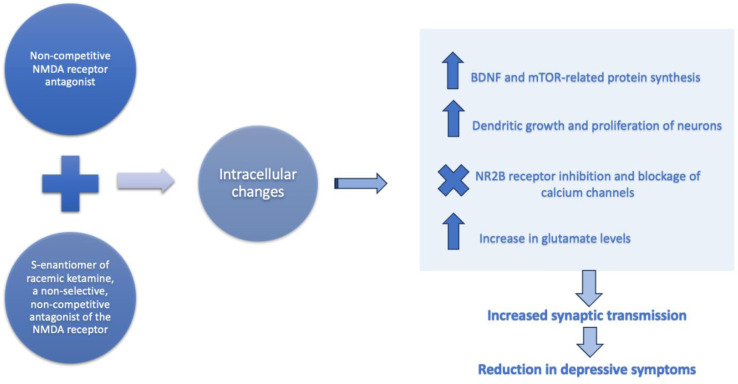
Ketamine antidepressant mechanism of action.

The main mechanism of ketamine is acting as a non-competitive blockade of the ion channel connected to the NMDA receptor complex. The glutamate surge is produced by the blockade of the NMDA receptors on GABA neurons, activating AMPA receptors [13]. After comparing antidepressant effects of other drugs with similar mechanisms of action such as memantine and seeing less efficacy, it is understood that this non-competitive blockade of the NMDA receptor is not the only mechanism to explain ketamine's antidepressant effects [13]. A proposed explanation is that the activated AMPA receptors then result in increased neuroplasticity along with higher levels of the associated protein, brain-derived neurotrophic factors with phosphorylation of tropomyosin receptor kinase B (TrkB) [13]. An additional proposal that has yet to be contradicted is attributed to its downstream activation of the mTOR pathway accompanied by the formation of spine synapses in the prefrontal cortex that was observed in rat models [13]. The mTOR pathway has impaired functionality in the prefrontal cortex in patients with MDD [16]. Furthermore, researchers were able to connect these 2 factors to ketamine's efficacy as an antidepressant by noting the return of depressive signs in the rats once they added a blockade of the mTOR pathway [13]. The recent approval of esketamine has been ground-breaking, given that the majority of antidepressants are monoamine oxidase inhibitors and take weeks while ketamine offers a novel mechanism of action by targeting glutamate receptors that work in a fraction of the time [10]. Figure 1 summarizes the proposed cellular mechanism.

### 3.4. Use of ketamine for depression and suicidality in terminal and cancer patients

Receiving a cancer diagnosis is a life-changing event that can cause a wide range of emotions from shock and disbelief to sadness, fear and anger. It is normal for cancer patients to experience a wide range of emotions throughout their journey, but when these emotions persist for a prolonged period of time, they may indicate depression. Depression and cancer have a complex and bidirectional relationship. On the one hand, depression can increase the risk of cancer incidence and mortality. On the other hand, cancer diagnosis and treatment can cause depression in certain patients. Depression is a common comorbidity in cancer patients with prevalence rates ranging from 10% to 40%.

The physical and emotional tolls that come with a cancer diagnosis can be overwhelming. Various studies show that the prevalence of suicidal ideation in patients with cancer ranges from 13.1% [17] to 34.3%[18]. Compared to the general population, the risk of suicide in cancer patients is doubled [19]. Additionally, the prevalence of completed suicide is increased in patients with cancer, particularly if they have also been diagnosed with clinical depression [20]. In patients with cancer classified as “severe”, the prevalence of suicidal ideation ranges from 19.1% to 24.5% [21],[22]. The prevalence of suicidal ideation with a specific plan is increased in patients with cancer and contributing factors on suicidality vary by age and gender [23],[24]. If a patient with cancer also has been diagnosed with a substance use disorder or other mental health disorder, the risk of suicide may be further increased [25]. Lastly, the prevalence of depression and suicidality in cancer patients may depend on prognostic factors such as the type of cancer, location, stage and treatment modality [26]–[28]. For example, patients with advanced cancer or those receiving palliative care may have a higher prevalence of depressive syndromes and/or suicidality than those with early-stage cancer or those receiving treatment. Table 2 summarizes several risk factors that have been identified for suicidality in terminal and cancer patients.

**Table 2.** Risk factors related to suicide in terminal patients.

**Risk Factors**
Family and personal history of suicide
Past psychiatric history (delirium, depression with profound hopelessness, psychotic features, impulsivity)
Uncontrolled pain or other symptoms of advanced disease
Cancer site (head and neck, lung, stomach and colorectal)
Limited social support
Advanced age
Male gender

Patients with both cancer and depression have been shown to have a reduced ability to cope with emotional distress, leading to a decreased quality of life, nonadherence to medical treatment and decreased survival rates [29],[30]. Additionally, depression is associated with an impaired immune response [29] and an increased perception of pain which may increase lengths of hospital stays, reduce drug efficacy and further decrease overall health outcomes [31]. Furthermore, a patient suffering from depression may delay seeking medical care for their cancer symptoms which may worsen their prognosis [32].

### 3.5. Overview of treatment for depression & suicidality in cancer and terminally ill patients

Much like any other medical diagnosis, treatment options for depression range from supportive psychotherapy, antidepressants and on rare occasions, ECT. These patients require an interdisciplinary treatment approach including management of pain, fatigue and somatic symptoms [33]. Therefore, the overarching goal of treating depression in cancer patients is to improve quality of life and at best, survival rate [34]. Like any terminal or cancer patient being managed for depression, other factors come into play when choosing one class of drug over the other including tolerability, side effect profile and patient preference [35].

Antidepressants can be a valuable tool in addressing cancer-related depression, as they can improve mood, reduce anxiety and help patients cope with the challenges of their disease. Studies have shown that antidepressants can be effective in managing depression in cancer patients with a reduction in symptoms ranging from 50% to 75%. Antidepressants can also help alleviate other symptoms of cancer, such as pain, fatigue and insomnia which can further improve a patient's quality of life. In addition, antidepressants have been shown to be safe and well-tolerated in cancer patients with few side effects reported.

Conventional use of antidepressants in terminal patients is not generally recommended given the delay in the onset of action of this. Moreover, depression scores suggest that antidepressants do very little to alleviate terminally ill patients of their depression [36]. Stimulants such as methylphenidate and dextroamphetamine have been found to be effective in treating depression in cancer patients. The use of stimulants as an antidepressant has been studied extensively and research has shown that they can improve mood, increase energy levels and reduce fatigue in cancer patients suffering from depression [37]. Overall, these medications can also have a positive impact on a patient's overall health, as depression has been linked to poor treatment outcomes and a higher risk of mortality in cancer patients.

### 3.6. Use of ketamine in treating depression & suicidality in cancer and terminally ill patients

Ketamine has also been studied for its antidepressant effects and is known to manage depression rather rapidly in terminally ill patients [38]. Although ketamine has been studied in the management of depression and found to be efficacious, it has been minimally studied in cancer patients receiving palliative care. While ketamine has shown promise in improving depressive symptoms, there are still many questions regarding its use in clinical practice. Table 3 summarizes the recommendations for clinical use of ketamine [9].

Ketamine has many properties that make it an interesting candidate for rapidly treating depression and anxiety in patients receiving hospice care [39]. A 28-day, open-label, proof-of-concept trial of daily oral ketamine administration was conducted to evaluate the tolerability. The study found that daily oral ketamine administration was well-tolerated and resulted in significant improvement in depression and anxiety symptoms in patients receiving hospice care [39]. Another study reported that a single oral dose of ketamine provided rapid and moderately sustained symptom relief for both depression and anxiety in patients receiving hospice care [40]. A retrospective chart review of 31 inpatients receiving hospice care who received ketamine for depression on a clinical basis was conducted and found that ketamine was effective in treating depression in patients receiving hospice care [41].

More recently, the INKeD-PC study also demonstrated robust antidepressant effects that were partially sustained with adequate safety and tolerability in these depressed patients with cancer receiving palliative care [42]. It has been shown to have rapid and effective anti-suicidal effects in depressed patients with suicidal ideation [43]–[45]. A single dose of ketamine has been shown to rapidly relieve acute suicidal ideation in cancer and palliative care patients [46]. Dosage recommendations should be tailored to the patient's specific needs and medical condition. Table 4 summarizes the details regarding ketamine/esketamine treatment in this population.

However, additional research on ketamine's long-term safety and its efficacy in reducing depression and suicide risk is needed before clinical implementation [43].

### 3.7. Safety and side effects of ketamine in cancer and palliative care patients

Overall, ketamine is considered safe in TRD and especially in patients experiencing suicidal ideations. Despite being a safe, effective and rapid treatment option for cancer patients battling depression, ketamine is ridden with side effects that are considered to sometimes outweigh its benefit. The most common side effects reported include dissociation, confusion, sedation, high blood pressure, dizziness, headache, blurred vision, anxiety, nausea and vomiting. Iglewicz et al described side effects based on his study to include disorientation, hallucinations, sedation, insomnia, delusions and anxiety. About 13% of study subjects experienced only one side effect including insomnia, delusions and anxiety and less than 30% of subjects experienced psychiatric symptoms. Overall, there were significantly more subjects (59%) who experienced no side effects [41]. Table 5 outlines major side effects reported in the literature.

**Table 3.**
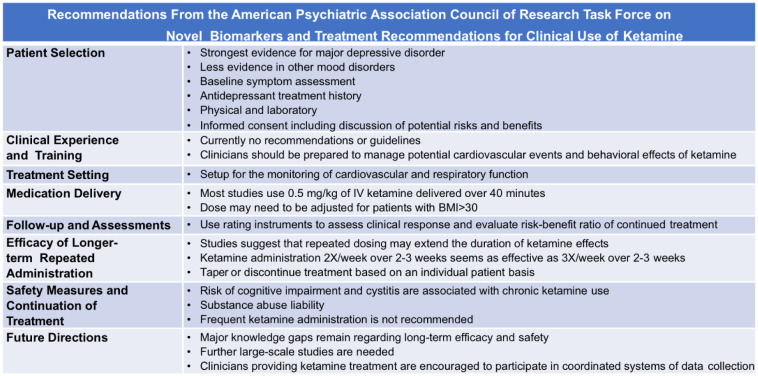
The recommendations from the American Psychiatric Association Council of Research Task Force on Novel Biomarkers and Treatment Recommendations for Clinical Use of Ketamine [9].

**Figure 2.**
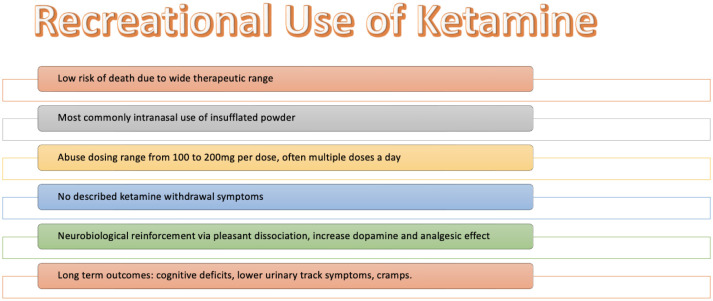
Recreational Use of Ketamine.

Despite its therapeutic benefits and positive outcomes, there have been concerns about the potential abuse or misuse of esketamine. (S)-ketamine has been found to have a higher binding affinity for NMDA receptors compared to (R)-ketamine which explains its greater anesthetic potency and undesirable psychotomimetic side effects [47]. Furthermore, (R)-ketamine appears to lack psychotomimetic side effects and potential for abuse, unlike (S)-ketamine [47]–[49]. These findings suggest that (R)-ketamine may be a safer alternative with lower abuse potential compared to (S)-ketamine. In terms of pharmacological profile, (S)-ketamine has been suggested to exhibit higher potential liability for abuse than (R)-ketamine [50]. Behavioral studies have also indicated that (S)-ketamine may have a higher abuse potential compared to (R)-ketamine [50]. Additionally, (S)-ketamine has been associated with acute psychotomimetic and dissociative adverse effects which may contribute to its abuse potential [48]. Studies have also found that the likability of esketamine and ketamine among recreational drug users is similar. This suggests that if esketamine were to be abused, it would likely have similar effects to ketamine. Real-world evidence from ketamine use supports the low potential for abuse and misuse of esketamine. In the general population, the abuse rate of ketamine has been found to be less than 0.2% and deaths due to ketamine are exceedingly rare. These findings suggest that the potential for abuse and misuse of esketamine is also likely to be low. Figure 2 summarizes other clinical aspects of recreational use of ketamine.

**Table 4.** Ketamine-esketamine treatment protocol.

Stage	Treatment protocol
Pretreatment Stage	Presentation for treatment Patient is NPO for 4 hours (solids), 2 hours (liquids) Evaluation with psychometric rating scales Montgomery Asberg Depression Rating Scale (MADRS) Quick Inventory of Depressive Symptomatology (QIDS) Insert IV or train with esketamine delivery device Vitals monitored throughout treatment IV: Mix 0.5 mg/kg of ketamine in 500 cc NS (dosing based on *ideal body weight*) IN: Select between 24 mg, 56 mg, 84 mg doses (typically start 56mg vs 84mg) Last “pre-briefing” / anticipatory guidance
Treatment Stage	During protocol – patient monitored by nursing Continuous pulse oximetry Continuous telemetry Blood pressure q15 minutes (IV), basline, 40 min, 2 hours (IN) Maintain a low stimulus environment (lights dimmed, quiet, soothing music)

**Table 5.** Side effects of ketamine.

Side Effect	Description
Dissociation	Occurs during treatment, often diminishes with repeated dosing.
BP and HR changes	Elevation in heart rate and blood pressure during treatment; patients counseled to take antihypertensives and antiarrhythmics on treatment days.
GI side effects	Nausea and vomiting during treatment; pre-medication with anti-emetics is helpful.
Other	Mild-headache and fatigue post treatment.

## 4. Discussion

Ketamine has demonstrated various benefits in the treatment of depression and suicidal ideations among patients with terminal illness. The drug has exhibited a capacity to diminish suicidal thoughts up to a week after treatment initiation with a notable reduction occurring within the first 24 hours [43]. In addition, ketamine has demonstrated a distinctive anti-suicidal effect which is separate from its general antidepressant characteristics [51]. Furthermore, ketamine has been proven to be harmless and advantageous in managing patients who are acutely suicidal or emotionally unstable in the short term [52]. Moreover, it has come to light that this psychotropic agent is effective in decreasing depressive symptoms and suicidal ideations in patients with treatment-resistant depression [53]. Additionally, ketamine has been proven to be effective in treating sudden-onset depression and active suicidal ideations in patients who have recently been diagnosed with cancer [44]. Finally, ketamine has been determined to be efficacious and well-tolerated in the treatment of chronic neuropathic pain and self-harming tendencies in children [54].

**Figure 3.**
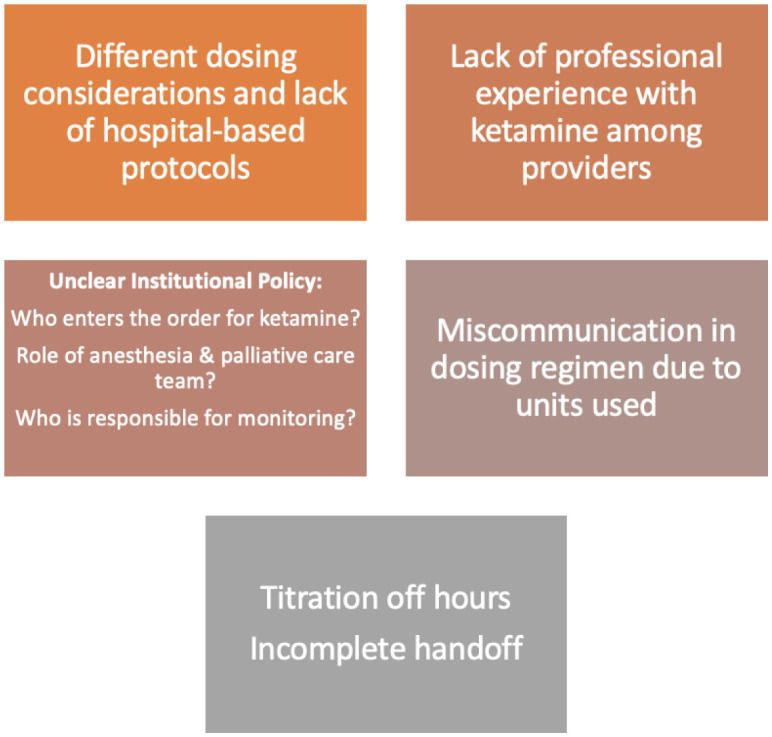
Practical obstacles of ketamine use in palliative care.

While ketamine has shown promise as a treatment for depression and suicidality in cancer and terminal patients, there are also several limitations and challenges associated with its use. One major limitation is the lack of long-term data on the safety and efficacy of ketamine for these patients [55]. Additionally, the optimal dosing and duration of treatment with ketamine for depression and suicidality in cancer and terminal patients is not yet established [38]. Another challenge is the potential for adverse effects, such as cognitive dysfunction, psychotomimetic effects and cardiovascular events [55]. Furthermore, the mechanism of action of ketamine in reducing suicidal ideation is not fully understood and the relationship between its effects on depression, anxiety and suicidal ideation is not clear [46]. Finally, there is a need for further research to establish the role and appropriate dosing of ketamine in cancer patients suffering from depression [38]. Figure 3 summarizes other practical obstacles of ketamine use in palliative care settings.

### 4.1. Future directions

Future directions for research on ketamine and depression/suicidality in cancer and terminal patients include the need for well-designed, randomized studies to establish the role and appropriate dosing of ketamine in this patient population. Further research is also needed to evaluate the long-term safety and efficacy of ketamine for these patients. Additionally, future studies should investigate the neural correlates of ketamine's anti-suicidal effects and its potential use as a crisis intervention in patients at suicide risk. Other future directions include investigating the optimal dosing and duration of treatment with ketamine for depression and suicidality in cancer and terminal patients as well as identifying clinical characteristics that predict symptom improvement with ketamine treatment. Finally, future research should aim to address the knowledge gaps about the use of ketamine in depression and develop novel, more effective and fast-acting antidepressants.

## 5. Conclusions

Due to the increased risk of suicide in cancer patients, there is a need for rapid-acting treatments for depression in this patient population [6]. Ketamine is used off-label for treatment resistant depression through its non-competitive blockade of the ion channel connected to the NMDA receptor complex on GABA neurons, activation of AMPA receptors and possible ability to increase neuroplasticity and cause downstream activation of the mTOR pathway [13]. Ketamine can be beneficial for depression and suicidality in cancer patients due to its rapid onset [8]. Esketamine, the S-enantiomer of ketamine, has been approved intranasally for treatment resistant depression [13]. Ketamine has been studied in terminally ill patients and found to be well-tolerated and improve depression and anxiety symptoms [38]–[41]. The INKeD-PC study found that ketamine had robust antidepressant effects in depressed patients with cancer receiving palliative care [42]. However, before prescribing ketamine, it is important to consider its side effects such as dissociation, BP and HR changes, nausea and vomiting and headache and fatigue [41]. Esketamine has a low potential for abuse and misuse when used as directed in clinical trials. Overall, ketamine and esketamine appear to be safe possible options for depression and suicide in cancer patients. Future research could include randomized controlled trials comparing the use of ketamine and esketamine to current first-line options for depression and suicide in cancer patients.

## Use of AI Tools Declaration

The authors declare they have used Artificial Intelligence (AI) tool (chat.openai.com) for generating an initial framework that guided the subsequent development of the manuscript. By employing the SciSpace Paraphraser, authors were able to enhance the clarity and readability of the content (e.g. discussion) without compromising its accuracy. It is important to note that while AI tools were employed in the planning stage of the manuscript, the content, analysis and interpretation of the research findings presented in the article were conducted by the authors themselves. The AI tool was not involved in the data collection, data analysis or drawing of conclusions. Authors reviewed and revised the paraphrased content to ensure it aligns with the context, requirements and ethics of academic writing.

## References

[1] Polsky D, Doshi JA, Marcus S (2005). Long-term risk for depressive symptoms after a medical diagnosis. Arch Intern Med.

[2] Fan CY, Chao HL, Lin CS (2018). Risk of depressive disorder among patients with head and neck cancer: A nationwide population-based study. Head Neck.

[3] Rasic DT, Belik SL, Bolton JM (2008). Cancer, mental disorders, suicidal ideation and attempts in a large community sample. Psychooncology.

[4] Honda K, Goodwin RD (2004). Cancer and mental disorders in a national community sample: findings from the national comorbidity survey. Psychother Psychosom.

[5] APA (2022). Diagnostic and statistical manual of mental disorders.

[6] Miller K, Massie MJ (2019). Oncology.

[7] Sullivan DR, Forsberg CW, Ganzini L (2016). Depression symptom trends and health domains among lung cancer patients in the CanCORS study. Lung Cancer.

[8] McIntyre RS (2010). When should you move beyond first-line therapy for depression?. J Clin Psychiatry.

[9] Sanacora G, Frye MA, McDonald W (2017). A consensus statement on the use of ketamine in the treatment of mood disorders. JAMA Psychiatry.

[10] Zhang Y, Ye F, Zhang T (2021). Structural basis of ketamine action on human NMDA receptors. Nature.

[11] Orhurhu VJ, Vashisht R, Claus LE (2023). Ketamine Toxicity.

[12] Rosenblat JD, Carvalho AF, Li M (2019). Oral ketamine for depression: a systematic review. J Clin Psychiatry.

[13] Dean RL, Hurducas C, Hawton K (2021). Ketamine and other glutamate receptor modulators for depression in adults with unipolar major depressive disorder. Cochrane Database Syst Rev.

[14] Andrade Chittaranjan (2017). Ketamine for depression, 3: Does chirality matter?. J Clin Psychiatry.

[15] Derakhshanian S, Zhou M, Rath A (2021). Role of ketamine in the treatment of psychiatric disorders. Health Psychol Res.

[16] Ignácio ZM, Réus GZ, Arent CO (2016). New perspectives on the involvement of mTOR in depression as well as in the action of antidepressant drugs. Br J Clin Pharmacol.

[17] Tang L, He Y, Pang Y (2022). Suicidal ideation in advanced cancer patients without major depressive disorder. Psychooncology.

[18] Sauer C, Grapp M, Bugaj TJ (2022). Suicidal ideation in patients with cancer: Its prevalence and results of structural equation modelling. Eur J Cancer Care (Engl).

[19] Manojna Konda, Rohan Sharma, Arya Mariam Roy (2019). Risk factors associated with suicide in patients with prostate cancer in the United States. J Clin Onc.

[20] Hopko DR, Armento ME, Robertson SM (2011). Brief behavioral activation and problem-solving therapy for depressed breast cancer patients: randomized trial. J Consult Clin Psychol.

[21] Leal-Hernández D A, Sandoval L, Palacios-Espinosa X (2014). Proposed scales for measuring suicidal ideation in adult cancer patients. Open J Med Psychology.

[22] Tanriverdi D, Cuhadar D, Ciftci S (2014). Does the impairment of functional life increase the probability of suicide in cancer patients?. Asian Pac J Cancer Prev.

[23] Men VY, Emery CR, Lam TC (2022). Suicidal/self-harm behaviors among cancer patients: a population-based competing risk analysis. Psychol Med.

[24] Hagezom HM, Amare T, Hibdye G (2021). Magnitude and associated factors of suicidal ideation among cancer patients at Ayder Comprehensive Specialized Hospital, Mekelle, Ethiopia, 2019: Cross-sectional study. Cancer Manag Res.

[25] Pitman A, Suleman S, Hyde N (2018). Depression and anxiety in patients with cancer. BMJ.

[26] Ristevska-Dimitrovska G, Stefanovski P, Smichkoska S (2015). Depression and resilience in breast cancer patients. Open Access Maced J Med Sci.

[27] Nakamura Y, Kanemoto E, Kajizono M (2017). Investigation of Mental Disorders in Lung Cancer Outpatients: A Retrospective Analysis. Yakugaku Zasshi.

[28] Tosic Golubovic S, Binic I, Krtinic D (2022). Risk factors and predictive value of depression and anxiety in cervical cancer patients. Medicina (Kaunas).

[29] Barrera I, Spiegel D (2014). Review of psychotherapeutic interventions on depression in cancer patients and their impact on disease progression. Int Rev Psychiatry.

[30] Huang RW, Chang KP, Marchi F (2022). The impact of depression on survival of head and neck cancer patients: A population-based cohort study. Front Oncol.

[31] Widiyono W, Setiyarini S, Effendy C (2019). Self-selected individual music therapy for depression during hospitalization for cancer patients: Randomized controlled clinical trial study. Indones J Cancer.

[32] Sherrill C, Smith M, Mascoe C (2017). Effect of treating depressive disorders on mortality of cancer patients. Cureus.

[33] Lloyd-Williams M, Dennis M, Taylor F (2004). A prospective study to determine the association between physical symptoms and depression in patients with advanced cancer. Palliat Med.

[34] Pinquart M, Duberstein PR (2010). Depression and cancer mortality: a meta-analysis. Psychol Med.

[35] Rodin G, Lloyd N, Katz M (2007). The treatment of depression in cancer patients: a systematic review. Supportive Care in Cancer.

[36] Lloyd-Williams M, Payne S, Reeve J (2013). Antidepressant medication in patients with advanced cancer--an observational study. QJM.

[37] Olin J, Masand P (1996). Psychostimulants for depression in hospitalized cancer patients. Psychosomatics.

[38] Stefanczyk-Sapieha L, Oneschuk D, Demas M (2008). Intravenous ketamine “burst” for refractory depression in a patient with advanced cancer. J Palliat Med.

[39] Irwin SA, Iglewicz A, Nelesen RA (2013). Daily oral ketamine for the treatment of depression and anxiety in patients receiving hospice care: A 28-day open-label proof-of-concept trial. J Palliat Med.

[40] Irwin SA, Iglewicz A (2010). Oral ketamine for the rapid treatment of depression and anxiety in patients receiving hospice care. J Palliat Med.

[41] Iglewicz A, Morrison K, Nelesen RA (2015). Ketamine for the treatment of depression in patients receiving hospice care: A retrospective medical record review of thirty-one cases. Psychosomatics.

[42] Rosenblat JD, deVries FE, Doyle Z (2023). A phase II, open-label clinical trial of intranasal ketamine for depression in patients with cancer receiving palliative care (INKeD-PC Study). Cancers (Basel).

[43] Wilkinson ST, Ballard ED, Bloch MH (2018). The effect of a single dose of intravenous ketamine on suicidal ideation: A systematic review and individual participant data meta-analysis. Am J Psychiatry.

[44] Fan W, Yang H, Sun Y (2017). Ketamine rapidly relieves acute suicidal ideation in cancer patients: a randomized controlled clinical trial. Oncotarget.

[45] Grunebaum MF, Galfalvy HC, Choo TH (2018). Ketamine for rapid reduction of suicidal thoughts in major depression: A midazolam-controlled randomized clinical trial. Am J Psychiatry.

[46] Ballard ED, Ionescu DF, Vande Voort JL (2014). Improvement in suicidal ideation after ketamine infusion: relationship to reductions in depression and anxiety. J Psychiatr Res.

[47] Yang C, Qu Y, Fujita Y (2017). Possible role of the gut microbiota–brain axis in the antidepressant effects of (R)-ketamine in a social defeat stress model. Transl Psychiatry.

[48] Chang L, Toki H, Qu Y (2018). No Sex-specific differences in the acute antidepressant actions of (R)-ketamine in an inflammation model. Int J Neuropsychopharmacol.

[49] Qu Y, Yang C, Ren Q (2017). Comparison of (R)-ketamine and lanicemine on depression-like phenotype and abnormal composition of gut microbiota in a social defeat stress model. Sci Rep.

[50] Bonaventura J, Lam S, Carlton M (2021). Pharmacological and behavioral divergence of ketamine enantiomers: Implications for abuse liability. Mol Psychiatry.

[51] Price RB, Mathew SJ (2015). Does ketamine have anti-suicidal properties? Current status and future directions. CNS Drugs.

[52] Abbar M, Demattei C, El-Hage W (2022). Ketamine for the acute treatment of severe suicidal ideation: double blind, randomised placebo controlled trial. BMJ.

[53] Vidal S, Gex-Fabry M, Bancila V (2018). Efficacy and safety of a rapid intravenous injection of ketamine 0.5 mg/kg in treatment-resistant major depression: An open 4-week longitudinal study. J Clin Psychopharmacol.

[54] Weber G, Yao J, Binns S (2018). Case report of subanesthetic intravenous ketamine infusion for the treatment of neuropathic pain and depression with suicidal features in a pediatric patient. Case Rep Anesthesiol.

[55] Zhu W, Ding Z, Zhang Y (2016). Risks associated with misuse of ketamine as a rapid-acting antidepressant. Neurosci Bull.

